# Understanding the nature of psychiatric comorbidity in migraine: a systematic review focused on interactions and treatment implications

**DOI:** 10.1186/s10194-019-0988-x

**Published:** 2019-05-09

**Authors:** Thomas Dresler, Salvatore Caratozzolo, Kaat Guldolf, Jana-Isabel Huhn, Carmela Loiacono, Triinu Niiberg-Pikksööt, Marta Puma, Giorgia Sforza, Anna Tobia, Raffaele Ornello, Gianluca Serafini, on behalf of the European Headache Federation School of Advanced Studies (EHF-SAS)

**Affiliations:** 1 Department of Psychiatry & Psychotherapy, University Hospital Tuebingen, Tuebingen, Germany; 2 LEAD Graduate School & Research Network, University of Tuebingen, Tuebingen, Germany; 3 Neurology Unit - Neurological and Vision Sciences Department, ASST Spedali Civili of Brescia, Brescia, Italy; 4 Department of Neurology, University Hospital Brussels, Jette, Belgium; 5 Praxis Gendolla, Specialized care for Psychiatry, Neurology, Psychotherapy and Pain Therapy, Essen, Germany; 6 Child Neuropsychiatry school, University of Palermo, Palermo, Italy; 7 Department of Neurology and Neurosurgery, University of Tartu, Tartu, Estonia; 8 Headache Centre & Neurocritical Care Unit, Department of Human Neurosciences, Sapienza - University of Rome, Viale dell’Università 30, 00185 Rome, Italy; 9 Child Neurology Unit, Department of Neuroscience and Neurorehabilitation, Headache Center, Bambino Gesù Children’s Hospital, IRCCS, Rome, Italy; 10 Child Neuropsychiatry Unit, ASL 3, Turin, Italy; 11 Department of Applied Clinical Sciences and Biotechnology, University of L’Aquila, L’Aquila, Italy; 12 Department of Neuroscience, Rehabilitation, Ophthalmology, Genetics, Maternal and Child Health (DINOGMI), Section of Psychiatry, University of Genoa, Genoa, Italy; 13 IRCCS Ospedale Policlinico San Martino, Largo Rosanna Benzi 10, 16132 Genoa, Italy

**Keywords:** Migraine, Psychiatric disorders, Comorbidity, Biological pathways

## Abstract

**Background:**

Migraine is a highly prevalent and disabling neurological disorder which is commonly linked with a broad range of psychiatric comorbidities, especially among subjects with migraine with aura or chronic migraine. Defining the exact nature of the association between migraine and psychiatric disorders and bringing out the pathophysiological mechanisms underlying the comorbidity with psychiatric conditions are relevant issues in the clinical practice.

**Methods:**

A systematic review of the most relevant studies about migraine and psychiatric comorbidity was performed using “PubMed”, “Scopus”, and “ScienceDirect” electronic databases from 1 January 1998 to 15 July 2018. Overall, 178 studies met our inclusion criteria and were included in the current review.

**Results:**

According to the most relevant findings of our overview, the associations with psychiatric comorbidities are complex, with a bidirectional association of major depression and panic disorder with migraine. Importantly, optimizing the pharmacological and non-pharmacological treatment of either migraine or its psychiatric comorbidities might help clinicians to attenuate the burden of both these conditions.

**Conclusions:**

The available data highlight the need for a comprehensive evaluation of psychiatric disorders in migraine in order to promote an integrated model of care and carefully address the burden and psychosocial impairment related to psychiatric comorbidities in migraine.

## Background

Both migraine and psychiatric disorders are prevalent and burdensome conditions challenging the health care systems worldwide [1–5]. These conditions show a large overlap [6, 7] and epidemiological studies suggest that patients with migraine – especially those with chronic migraine (CM) and migraine with aura – are at increased risk for major depression, anxiety, or suicidal behavior when compared to subjects without migraine (e.g., [8–10]). Besides, according to a recent large genome-wide association study, when compared to other neurological disorders, migraine showed a higher genetic correlation with psychiatric disorders suggesting common genetic bases or pathways [11]. The comorbidity between migraine and psychiatric disorders is highly relevant in the clinical practice, as it might influence both the response to treatment and likelihood to achieve remission [6]. Therefore, an interdisciplinary approach using pharmacological and non-pharmacological treatment strategies aimed to manage both migraine and comorbid psychiatric disorder is essential.

The comorbidity between migraine and psychiatric disorders presumably implies multiple causes, including either unidirectional causal explanations, but even shared environmental and/or genetic risk factors, and their interaction at multiple levels [12]. All the mentioned aspects need to be carefully considered regarding the diagnostic and therapeutic implications related to migraine comorbidity.

This systematic review will provide an updated and comprehensive overview of the current literature focusing on the comorbidity of migraine with depression, bipolar disorder (BD), anxiety disorders, post-traumatic stress disorder (PTSD), and other psychiatric disorders, including personality, substance use, and somatoform disorders, outlining the general findings, potential mechanisms of association, and implications for migraine treatment.

## Materials and methods

**Fig. 1:**
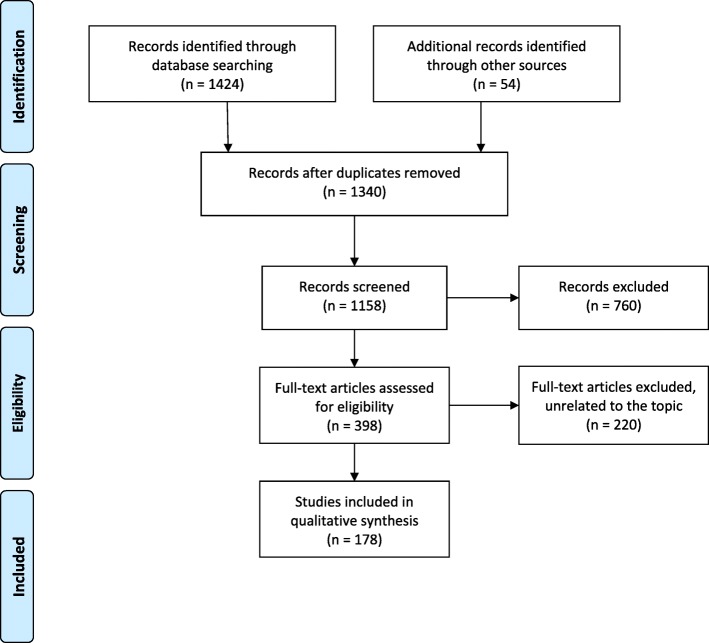
Flowchart of study selection. Figure 1 includes all the relevant steps and main results of the literature search upon the main topic. The most relevant studies have been selected in accordance with the PRISMA guidelines. Specifically, observational studies, clinical trials, open label studies, systematic reviews, guidelines, commentaries, editorials, and letters to editors focusing on the review topic were included

To achieve a high standard of reporting, we selected the most relevant studies in accordance with the PRISMA guidelines [13]. We included observational studies that explicitly and clearly report the adopted definitions of migraine and its psychiatric comorbidities; we also included clinical trials, open label studies, systematic reviews, guidelines, commentaries, editorials, and letters to editors focusing on the review topic. When a title/abstract appeared to describe a study eligible for inclusion, the full-text article was carefully analyzed to evaluate its relevance for our systematic review. Eligible papers had to be written in English and published from 1 January 1998 to 15 July 2018 on the following 3 major electronic databases: PubMed, Scopus, or ScienceDirect. The following search string was used in all databases: (“Headache” OR “migraine”) AND “comorbidity” AND (“psychiatric disorders” OR “substance abuse” OR “personality disorders” OR “major affective disorders” OR “bipolar disorder” OR “unipolar disorders” OR “psychotic disorders” OR “psychoses” OR “suicid*” OR “anxiety” OR “schizophrenia” OR “depression”). The reference lists of the retrieved articles were also screened to find eligible studies not covered by the above-mentioned search string. Two Reviewers (MP and GSf) conducted the literature search and independently screened titles and abstracts; later, they retrieved and selected full-text articles. Disagreements among these two Reviewers were solved by consensus. The relevant steps and main results of the literature search are shown in Fig. 1.

## Major depression

Major depressive episodes are characterized by periods of at least two weeks with symptoms including depressed or irritable mood, decreased interest or pleasure in most activities, significant weight change, change in sleep (insomnia or hypersomnia), change in activity (psychomotor agitation or retardation), fatigue or loss of energy, guilt/worthlessness, diminished ability to think or concentrate or more indecisiveness, and suicidality [14].

Depression is almost twice as frequent in patients with migraine when compared to the general population. The prevalence estimates in migraine vary across countries, from 6.1% to 73.7% (e.g., [15–17]) while the prevalence odds ratios vary from 0.8 to 5.8 (see Table 1). Such differences may be mainly due to different sex, age, and ethnic composition of study samples, as well as from different psychometric instruments aimed to assess migraine and depression [18]. Comorbid major depression is linked to more frequent and disabling headache [19]. Notably, the risk of suicide attempts is particularly higher in migraine patients with comorbid anxiety and depressive symptoms [20].

**Table 1:** Quantitative association between migraine and psychiatric comorbidities in observational studies. Studies reporting the proportions of comorbidities (first column) may not coincide with those reporting the effect sizes of associations (fourth column)

Comparison	Proportion of comorbidity in migraine (%)	Proportion of migraine in comorbidity (%)	No. of studies with positive association/ total studies	Effect size range	Reported potential confounders (no. of studies)
Episodic migraine vs no migraine					
Depression [15, 17, 22, 43, 46, 73, 74, 82, 131, 161, 165–179]	5.6 to 73.7	9.9 to 55	13/14 cross-sectional 4/4 case-control1/1 prospective cohort1/1 retrospective cohort [17, 22, 46, 55, 73, 74, 131, 161, 167, 169, 171–178, 180]	OR 0.8 to 5.8; HR 2.75; PR 2.7	Age (14), sex (16), education (9), income (4), residence area (2), marital status (2), race (1), smoking (1), urbanization level (1), self-rated health (1), sleep habits (1), high blood pressure (1), cervical pain (1), low back pain (1), asthma (1)
Bipolar disorder [15, 17, 43–46, 168, 181–184]	0.99 to 5.4	15.7 to 55.3	4/5 cross-sectional [15, 17, 46, 181, 183]	OR 0.9 to 3.7	Age (3), sex (3), education (2), income (2), marital status (1), residence (1), urbanization level (1)
GAD [72, 82, 101, 161, 165, 166, 170, 173, 176]	13.2 to 76.4	-	4/5 cross-sectional [72, 101, 161, 173, 176, 182]	OR 2.55 to 5.84	Age (5), sex (5), race (3), education (3), family income (1), marital status (2), smoking (1)
Panic disorder [15, 17, 72, 76, 77, 161, 165, 166, 170, 173]	0.58 to 61.3	61.1	2/3 cohort6/7 cross-sectional [15, 17, 23, 72, 76, 147, 161, 165, 170, 173]	OR 1.23 to 9.6; HR 3.55	Age (4), sex (4), race (2), education (2), income (3), marital status (2), urbanization level (1)
Simple phobia [161]	29.1	-	1/1 cross-sectional1 prospective [23, 161]	OR 1.66 to 2.43	Age (1), sex (1)
Social phobia [17, 161, 166]	6.7 to 27.0	-	1/2 cross-sectional1/1 prospective [17, 23, 161]	OR 1.45 to 14.3	Age (1), sex (1), marital status (1)
OCD [15, 72, 165, 166]	0.18 to 5.6	-	1/2 cross-sectional [15, 72]	OR 2.16 to 3.52	Age (2), sex (2), race (1), education (1), family income (2), marital status (1), SSRI use (1), urbanization level (1)
PTSD [176, 185]	21.5 to 25.7	-	2/2 cross-sectional [176, 185]	OR 1.75 to 3.07	Sex (2), anxiety (2), depression (2), age (1) marital status (1), race (1), education (1), smoking (1), drug/alcohol abuse (1)
Eating disorders [160]	-	22.0	0/1 case-control [160]	OR 2.0	Clustered sampling including depression
Recreational substance abuse [17, 161, 176]	2.3 to 64.5	-	0/3 cross-sectional [23, 161, 176]	OR 0.83 to 1.59	Age (3), sex (3), marital status (2), education (2), race (1), smoking (1)
Chronic migraine with or without medication overuse vs no migraine					
Depression [9, 15, 73, 74, 78, 178, 186]	11.0 to 57.0	-	3/4 cross-sectional,1/1 retrospective cohort [9, 15, 73, 74, 178]	OR 0.8 to 6.4; RR 5.83	Age (4), sex (3), education (2), income (2), urbanization level (1), deprivation score (1)
Bipolar disorder [15]	2.35	-	1/1 cross-sectional [15]	RR 1.90	Age, sex, income, urbanization level
GAD [72, 78, 101, 186]	6.8 to 41.8	-	0/1 cohort, 2/2 cross-sectional [72, 78, 101]	OR 6.99 to 13.18	Age (1), sex (1), race (1), education (1), income (1), marital status (1)
Panic disorder [15, 72, 186]	1.37 to 12.5	-	1/1 cohort, 1/1 cross-sectional [15, 72]	OR 2.85 to 3.98	Age (2), sex (2), race (1), education (2), income (2), marital status (1)
Simple phobia	-	-	-	-	-
Social phobia [78, 186]	2 to 3.4	-	-	-	-
OCD [15, 78, 186]	0.11 to 1.1	-	0/1 cross-sectional [15]	RR 1.25	Age (1), sex (1), income (1), urbanization level (1)
PTSD [78, 186]	1 to 2.3	-	-	-	-
Eating disorders [9, 186]	0 to 0.8	-	1/1 cross-sectional [9]	OR 1.48	Age, sex, deprivation score
Recreational substance abuse [9, 15, 186]	0 to 2.4	-	0/1 cross-sectional [9]	OR 0.92	Age, sex, deprivation score
Chronic migraine with or without medication overuse vs episodic migraine					
Depression [15, 18, 187]	CM: 5.7 to 39.0; EM: 2.4 to 17.24	-	1/2 cross-sectional1/1 retrospective cohort [15, 18, 187]	OR 2.00 to 6.39; RR 1.88	Age (3), sex (2), education (1), income (2), urbanization level (1)
Bipolar disorder [15]	CM: 2.35;EM: 0.99	-	1/1 cross-sectional [15]	RR 1.81	Age, sex, income, urbanization level
GAD [8, 72, 186]	CM: 6.8 to 41.8; EM: 9.8 to 23.1	-	1/1 cohort, 1/1 cross-sectional [8, 72]	OR 6.0 to 6.99	Age(1), sex (1), race (1), education (1), income (1), marital status (1)
Panic disorder [8, 15, 72, 186]	CM: 1.17 to 24.4; EM: 0.58 to 7.7	-	2/2 cohort,1/1 cross-sectional [8, 15, 72]	OR 1.54 to 12.1	Age (2), sex (2), race (1), education (2), income (2), marital status (1)
Simple phobia	-	-	-	-	-
Social phobia [8, 186]	CM: 3.4 to 34.1; EM: 0.8 to 12.2	-	1/1 cohort [8]	OR 4.3	-
OCD [15, 186]	CM: 0.15 to 1.1; EM: 0.18 to 2.3	-	0/1 cross-sectional [15]	RR 0.94	Age (1), sex (1), income (1), urbanization level (1)
PTSD [186]	CM: 2.3;EM: 0	-	-	-	-
Eating disorders [186]	CM: 0;EM: 0.8	-	-	-	-
Recreational substance abuse [8, 15]	CM: 0.15 to 43.9; EM: 0.04 to 14.6	-	1/2 case-control [8, 15]	OR 2.30 to 7.6	Age (2), sex (2), income (1), urbanization level (1)

*CM* chronic migraine, *EM* episodic migraine, *GAD* generalized anxiety disorder, *HR* hazard ratio, *OCD* obsessive-compulsive disorder, *OR* odds ratio, *PR* prevalence ratio, *PTSD* post-traumatic stress disorder, *RR* relative risk

### Mechanisms potentially involved in the comorbidity

Given the potential explanations of the comorbidity between migraine and major depression [12], studies indicate the existence of a bidirectional relation [21]. For instance, a cohort study found that the presence of each disorder (both migraine or depression) enhanced the risk for a first onset of the other [22], whereas in an older sample the presence of depression did not predict the onset of migraine [23]. Both migraine and depression show a specific heritability of about 40–50% having a polygenic background [24]. Twin studies suggest that about 20% of the variability in both migraine and depression can be attributed to shared genes with a bidirectional pattern [25, 26].

The serotonin (5-HT) system plays a crucial role in the association between migraine and depression. Indeed, patients with migraine have increased ictal 5-HT concentrations and decreased interictal 5-HT plasma levels, suggesting that a chronically reduced interictal 5-HT availability may predispose to cortical spreading depression and increased sensitivity of trigemino-vascular pathways [27]. Besides, a polymorphism in the 5-HT transporter gene has been linked to migraine as well as depression [28]. In addition, the migraine abortive drugs triptans usually act as 5-HT agonists and even selective serotonin reuptake inhibitors (SSRIs) may be used in preventing migraine, even if they are not a first-line preventive treatment and not recommended by existing guidelines.

A second possible key player in the association between migraine and depression is the dopaminergic system, as a dopamine D2 receptor genotype is significantly associated with comorbid migraine with aura, depression, and anxiety [29].

A study found significantly lower GABA cerebrospinal fluid (CSF) levels in CM patients with depression when compared to those without, suggesting that GABA is a possible mediator of the association between CM and depression [30]. A further possible link between depression and CM may be represented by the shared involvement of the hypothalamic-pituitary adrenal (HPA) axis [31]. Specifically, an imbalance between pro-inflammatory and anti-inflammatory cytokines resulting in abnormal increased pro-inflammatory cytokines levels has been hypothesized as a possible link between depression, migraine, obesity, and the progression from episodic migraine (EM) to CM, with underlying dysfunctions in tryptophan metabolism and serotonergic activation of the HPA axis [32]. This further points towards the potential role of the 5-HT system in the association between migraine and depression, suggesting multiple neural mechanisms interacting in that association.

Recent neuroimaging studies showed that specific pain-modulating brain areas, including the amygdala, anterior cingulate cortex, and periaqueductal gray show functional and structural alterations in both migraine and affective disorders, suggesting a common matrix underlying these conditions [33]. This would imply a dysfunction of a “neuro-limbic” pain network underlying migraine, in line with the findings from the current literature showing that the presence of anxiety-depressive symptoms influence the clinical presentation of migraine [34].

Finally, according to a psychological point of view, specific shared vulnerabilities between migraine and depression exist. Stress is a migraine trigger [35] and a risk factor of migraine chronification [36], but it also has a pivotal role in inducing major depression [33]. In addition, broad and unspecific personality traits, mostly neuroticism, have been implicated in the comorbidity between depression and migraine [37].

### Implications for treatment

The assumed bidirectional influence and the shared mechanisms underlying migraine and major depression could be used in a beneficial synergistic way when treating patients.

For instance, there is evidence that in patients with CM and comorbid depression, the prophylactic use of onabotulinumtoxin A significantly reduces headache as well as depressive and anxiety symptoms [38, 39]. Similarly, cognitive-behavioral therapy (CBT) improved headache symptoms, depression, anxiety, and quality of life of patients with migraine and/or tension-type headache and comorbid depression – with improvements that were maintained for at least 4 months [40]. Migraine guidelines recommend the use of amitriptyline, a tricyclic antidepressant, for migraine prophylaxis, which should be preferred when a comorbid depression has been diagnosed [41, 42]; however, it should be noted that the amitriptyline doses required for the treatment of migraine are lower than those used to treat depression [42]. Conversely, caution is needed regarding the use of flunarizine and beta-blockers to prevent migraine as they may be contraindicated in the presence of depression. Comorbid major depression is a complex and more difficult to treat clinical condition; yet, these patients do respond well to headache treatment, also resulting in an improved quality of life [19]. Hence, treating both disorders adequately may result in symbiotic treatment outcomes, preventing the development of chronification [42].

## Bipolar disorder

BD is characterized by a periodic course of depressive episodes and episodes with exceptionally increased mood (mania or hypomania). It is commonly divided into BD type I (at least one manic or mixed episodes) and BD type II (at least one hypomanic, but no full manic phase), with BD I usually impairing more severely the individual functioning [14].

Patients with BD display an increased prevalence of migraine that can reach up to 55.3% (Table 1), although – as stated above in the case of major depression – rates may considerably vary across countries due to several factors. Migraine prevalence seems to be higher in BD II than BD I [43, 44], and mostly migraine precedes the onset of BD [45]. In a population-based study, the prevalence of migraine was higher in subjects with both manic and depressive episodes than in those with depressive episodes only [46]. The available data suggest that BD has a more severe course when it is comorbid with migraine [47].

### Mechanisms potentially involved in the comorbidity

BD shows the highest heritability in the group of affective disorders, with a consistent overlap with migraine. The available literature data show that a positive family history of BD is a consistent risk factor for migraine [43, 47, 48], pointing at towards a possibly shared hereditary basis. Furthermore, based on a genome-wide linkage study [49] and association study [50], some shared genetic vulnerabilities may be supposed. Overall, multifactorial polygenetic mechanisms seem to confirm the existence of the comorbidity between migraine and BD.

As already stated above for comorbid depression, several neurotransmitter systems have been hypothesized to be involved in BD and comorbid migraine, with studies suggesting a dysfunction in serotonergic [27, 51, 52], dopaminergic [53, 54], and glutamatergic pathways [55, 56]. Furthermore, rather at the cellular level, alterations in specific sodium and calcium ion channels have been found in both migraine [57–59] and BD [60–62], a finding which might explain the common action of anti-epileptic drugs such as valproate in both disorders [63–65]. Finally, as in the case of major depression, even for BDy pro-inflammatory cytokines might play a role in determining the migraine-BD comorbidity [66].

### Implications for treatment

Among the available treatments with documented stabilizing properties in BD, valproate and topiramate have also been proven effective in the management of migraine [65] and there is some evidence suggesting the effectiveness of lamotrigine (which is only approved for the management of depressive recurrences in bipolar depression) for migraine prevention ([63], but also see [67]); the shared action of those drugs might point to a similar pathophysiology underlying BD and migraine. In addition to specific psychoactive medications, CBT [68] and particularly social rhythm therapy – a variant of interpersonal psychotherapy aimed at stabilizing endogenous circardian rhythms – were effective for the treatment of both BD and migraine [69, 70].

Conversely, the use of SSRIs and even more serotonin–norepinephrine reuptake inhibitors (SNRIs) is associated with the risk of exacerbating mania or initiating a more rapid cycling course in BD [71]. As migraine usually precedes the BD diagnosis [44], a switch into manic episodes might be precipitated by antidepressants aimed to treat migraine or first symptoms of depression. This underlines a considerable risk for misdiagnosis and mistreatment in comorbid patients.

## Anxiety disorders

Table 1 shows the quantitative data regarding the association between migraine and anxiety. Notably, the prevalence of anxiety increases with migraine frequency [72, 73], suggesting a ‘dose-response’ effect; the comorbidity between migraine and anxiety disorders is also enhanced by the presence of medication overuse [74] and concurrent depression [75]. As for major depression, the risk of suicide attempts is increased in patients with migraine and anxiety disorders [20].

### Panic disorder

Panic disorder (PD) is characterized by unexpected recurrent panic attacks, accompanied by physical symptoms such as sweating, trembling, palpitations, dizziness, chest pain, the fear of going crazy or dying, often co-occurring with agoraphobia [14]. When compared to individuals without migraine, patients with migraine are 1.2 to 9.6 times more likely to be diagnosed with PD (e.g., [76]) (see Table 1). According to the current literature, PD occurs earlier in patients with migraine as compared to those without [77]. However, the association between the two conditions is likely to be bidirectional, with the influence being primarily from headaches to PD although a weaker, yet significant influence was observed in the opposite direction [76]. The prevalence of PD is about 2–3 times higher in CM than in EM [78].

### Phobic disorders

Phobic disorders include specific phobia (fear of objects or situations) and social phobia (fear of socially relevant interactions) [14]. Evidence regarding the comorbidity between phobias and migraine is scarce [16, 79]. There seems to be an overlap regarding specific avoidance behaviors in migraine and phobias, which led some researchers to introduce the term ‘cephalalgiaphobia’, which may be linked to the risk of transformation to CM and medication-overuse headache (MOH) [80, 81]. A core feature of phobic-avoidant disorders is anticipatory anxiety, which may be the reason why some patients take hold of analgesics in the least warning of pain, eventually leading to a vicious circle of headache and medication overuse.

### Generalized anxiety disorder

Generalized anxiety disorder (GAD) is characterized by the presence of pervasive anxiety and repetitive worries about specific events [14]. The prevalence of GAD is higher in subjects with migraine than in those without migraine (see Table 1) [82]. If migraine is comorbid with depression and anxiety, patients tend to suffer from more severe migraine attacks, respond poorly to commonly available treatments, and are at increased risk of developing MOH [82–86]. Besides, the presence of GAD seems to precede migraine diagnosis which may have important treatment implications [87].

### Mechanisms potentially involved in the comorbidity

The bidirectional association between migraine and PD suggests that shared genetic or environmental factors might be involved in the comorbidity of PD with migraine and other severe headaches [76, 85]. Migraine and PD are likely to share an altered autonomic regulation. A further possible mechanism of association is somatization, which is typically found in patients with PD and might increase the prevalence of somatic symptoms, including migraine headaches [84].

In a rat model of CM, researchers found a high prevalence of anxiety- and depression-like symptoms, which could be reduced by a low-dose amitriptyline administration. Moreover, CM was associated with lower prefrontal 5-HT and dopamine levels. Translating these findings to humans, alterations in these neurotransmitter systems seem to contribute to both CM and anxiety [88].

Anxiety-depression symptoms in migraine may be linked to higher migraine trigger susceptibility. Here, central sensitization in migraine patients might be modulated and enhanced by comorbid anxiety-depression symptoms, increasing the risk of transformation to CM [89]. Such mechanism could be able to explain the prevalence of more severe headache or CM in anxiety.

On the neural level, anxiety might directly influence migraine symptoms acting on relay trigeminovascular thalamic neurons transmitting headache-related nociceptive signals, which are modulated by several excitatory and inhibiting input fibers. Surprisingly, the absence of calcitonin gene-related peptide (CGRP)-containing fibers around the thalamus indicates that CGRP is presumably not acting here, but more on a neurohormone level [90]. Finally, a small Chinese study in MOH patients indicates that headache and anxiety may be linked to changes in hippocampal volume [91].

Evidence regarding the comorbidity between migraine and anxiety disorders is mainly focused on shared neurotransmitter systems, primarily serotonergic dysfunction, which anxiety disorders share with depression. Other potential mechanisms involve ovarian hormone fluctuations, HPA axis dysregulation, and shared genetic influences [92]. Migraine and anxiety have been associated with the serotonin transporter gene 5-HTTLPR polymorphism and the C/C NcoI polymorphism within the dopamine receptor D2 gene [29, 93, 94]. However, there are association studies that did not find an association between the migraine-PD comorbidity and dopamine receptor genes [95]. This again points towards a multifactorial pattern of association.

A Dutch twin study found that anxious depression shared the heritability pattern of migraine, suggesting a bidirectional causal relation [25], while another Dutch study stressed the importance of anxiety and depression in pain disorders and suggested that anxiety and depression explain a substantial amount of the migraine comorbidity with other pain conditions [92].

Interoceptive conditioning, fear of pain, anxiety sensitivity, and avoidance behaviors have been considered as vulnerability factors for migraine and PD in their development and maintenance. Interactions between these variables warrant further longitudinal studies to elucidate etiological trajectories and pathophysiological mechanisms [94].

### Implications for treatment

A retrospective study found that the treatment of PD with antidepressant medications (SSRIs, tricyclic antidepressants [TCAs]) may may not only act beneficially on PD but also on comorbid migraine [77]. This finding hints towards a prophylactic effect, which could result in a win-win-solution for the patients.

Options for treating comorbid migraine and anxiety disorders include anticonvulsants [67, 96]: pregabalin, which is used for GAD, has been suggested as a useful alternative prophylaxis in CM according to one open-label study [97], while topiramate, which is used for social phobia, has been suggested as a useful treatment alternative for those who do not respond to or cannot tolerate SSRIs [98]. The antiglutamatergic effect of lamotrigine on migraine with aura and GABAergic effect of topiramate and valproate on migraine without aura might act on the neural alterations implied in both depression and anxiety [99].

Teaching about dysfunctional avoidance patterns in migraine, including excessive painkiller intake, may help patients to get insights into their avoidance of migraine triggers [100]. Henceforth, psychological interventions may be important to prevent medication overuse. In addition, treatment of phobias in CM may lead to lower anxiety and depression levels as well as better quality of life [79].

Considering that anxiety increases the likelihood to develop migraine, it is therapeutically important to notice subthreshold symptoms. Associations between subthreshold anxiety and primary headache have been described and subthreshold anxiety showed significantly higher ORs for all headaches, migraine, and CM [101, 102].

The careful screening of children, adolescents, and young adults who suffer from migraine for both anxiety and depression, and vice versa, might result in better treatment options and improved long-term outcomes for the patient [103, 104]. Besides, the adequate screenings may reveal underlying or subclinical psychiatric disorders [89].

An integration of behavioral strategies for managing comorbid conditions into existing treatment protocols pursues to modify dysfunctional behaviors and cognitions [36].

Recognition of comorbid psychiatric disorders is also advisable to prevent unwanted drug effects in comorbid patients and to permit drugs effective for both disorders [105], aiming to prevent headache worsening, chronification, or medication overuse.

CBT strategies are usually aimed at modifying dysfunctional behaviors, thoughts, and feelings that incidentally maintain both depression and anxiety. This may enhance adherence to pharmacotherapy, helping to minimize the potential for headache chronification. Some behaviors associated with anxiety include dysfunctional avoidance patterns for which CBT may be really helpful in the clinical practice [36, 106, 107].

A multidisciplinary treatment approach in migraine (i.e., combining both pharmacological and psychological approaches with other strategies such as physiotherapy) (see e.g., [108]) should be aimed at excluding conflicts, aggression as well as factors that contribute to anxiety. The multidisciplinary treatment approach should be personalized and take into account both the patients’ usual behavior and environment, thus helping patients to recover their stability and avoid the anxious anticipation of the next attack [109].

## Stress and post-traumatic stress disorder

The relation between stress and migraine is bidirectional and may be direct or indirect. Patients with migraine report higher stress levels when compared to healthy controls (e.g., [109]), and stressors are usually reported as migraine triggers [84, 110–115]. Conversely, migraine itself acts as a stressor resulting in a vicious circle with a strong impact on important individual domains such as work and social functioning [116]. Stress exposure might also mediate the association between migraine and other psychiatric comorbidities, including major depression [112], and is implied in the transformation of headache into chronic headache [117, 118].

The development of PTSD mandatorily depends on the direct or indirect exposure to traumatic life events and is characterized by intrusive symptoms, avoidance, and negative alterations in both cognitions and mood [14]. PTSD is related to the development of pain disorders [119], with a higher prevalence in patients with migraine, and mostly CM, compared to healthy controls [120]. Notably, the available studies found a higher incidence of childhood abuse in migraine patients with BD or depression as compared to those with migraine only [110, 121]. There is also evidence suggesting that PTSD, but not the mere exposure to a traumatic event, is correlated with migraine [122], while in the absence of definite PTSD, only repetitive (≥3) traumatic events enhance the risk for migraine [123–126]. Besides, CM is associated with a higher susceptibility for PTSD compared to EM; indeed, patients with CM were more influenced by traumatic events, as evident in more avoidant and re-experiencing symptoms when compared to patients with EM [127].

PTSD is more frequent among patients with CM when compared to those with chronic tension-type headache [128], suggesting that the association is specific to migraine. Moreover, PTSD with comorbid depression was associated with a higher risk of migraine chronification when compared to depression alone [120]. In line with those findings, an Italian study found that major traumatic events were associated with CM and MOH [129]. Not surprisingly, patients with migraine and comorbid PTSD report greater headache-related disability and quality of life impairment than those with migraine only [120, 122, 130].

### Mechanisms potentially involved in the association

The higher prevalence of stress and stress-related disorders in patients with migraine compared to non-migraineurs might be explained by the central sensitization theory, which postulates a stress-induced abnormal activation of the trigeminal nucleus caudalis, hypothesized to be involved in pain processing [131]. Stress-induced mechanisms acting on underlying genetic and epigenetic vulnerability are able to modify neural circuits, neurotransmitter balance, and autonomic and endocrine responses [132]. The stress response seems to be dysfunctional even in children with migraine [111, 133]. The load of stress and repeated migraine attacks may impair the allostasis of the brain, resulting in a dysregulated neural and endocrine response, i.e., the “allostatic load” [134].

Serotonin is likely a mediator of the relation between migraine and stress [27, 90], even if the available data did not show a specific mediation of serotonin in the association between PTSD and migraine. Stress plays a major role even in the trigemino-vascular system, whose activation is provoked from prolonged stress through the action of the HPA axis [135].

A theory explaining the higher prevalence of migraine in PTSD is the so-called “limbically augmented pain syndrome” [136]. According to that theory, if the normal arousal induced by pain becomes chronic, the brain fails to adjust adequately, causing an abnormal endocrine response as well as permanent changes in the limbic system. This phenomenon is shared by both migraine and PTSD, in which the exposure to major traumatic events impairs the normal limbic response. Another possible explanation of the comorbidity between stress and migraine is that chronic stress provokes a state of prolonged inflammation leading to a damage and substantial modifications on some sensitive specific brain areas, even implied in pain perception [132, 136–139].

### Implications for treatment

Individuating the presence of PTSD in patients with migraine is crucial for the management of migraine, considering that treating PTSD alone could improve the sense of well-being, and significantly reduce pain and disability in patients with migraine [120].

Controlling the amount of stress may be crucial for headache management [112] as migraine may also be associated with a dysfunctional coping style [140]. Adolescents with CM are more prone to adopt passive coping strategies to face stress, especially when migraine is associated with depression [141]. In this context, CBT is increasingly gaining consensus as a fundamental part of migraine management. CBT may be really focused on attack prevention [106]. The aim of this treatment approach is to change dysfunctional behaviors which are significantly involved in maintaining depression and anxiety [36] and comprises stress management and coping [106]. The highest benefits of CBT are observed when combined with pharmacological treatment [117, 142].

## Other psychiatric comorbidities

### Personality traits/disorders

Current evidence suggests that there are not dominant personality profiles among migraine patients; however, personality disorders seem to complicate headache treatment [117, 143].

A meta-analysis of ten observational studies showed that children with migraine tend to show more internalizing (“anxious, inhibited”) and externalizing (“aggressive and antisocial”) behaviors, as measured by the Child Behavior Checklist (CBCL), than healthy children, even if the difference was more evident for internalizing behavior [144]. According to a systematic review, children with migraine tend to show more somatic complaints and internalizing behaviors than healthy controls, which might be attributed to chronic pain rather than to psychological dysfunctioning [145].

Compared to healthy controls, females aged 18–65 years with a migraine had higher scores in the Harm Avoidance (HA) and Persistence (P) sections and lower scores in the Self-Directness sections of the Temperament and Character Inventory [146]. A population-based study performed among females aged 40–74 years found no association between lifetime migraine and personality traits or psychiatric disorders; however, in elderly females (60–74 years), the risk for active migraine was significantly and consistently associated with a history of major depression, higher levels of stress susceptibility and somatic trait anxiety [147].

An Italian multi-center study performed in tertiary headache clinics applied the Minnesota Multiphasic Personality Inventory (MMPI-2) and found – as compared to healthy controls – that patients with MOH and episodic headache, respectively, scored higher in the so-called ‘neurotic scales’ (i.e., Hypochondriasis, Depression, Hysteria) and lower in the Ego Strength and Dominance scales; besides, patients with MOH had higher scores in the Hypochondriasis and Health Concerns scales when compared to those with episodic headache [140]. Moreover, patients with CM and MOH did not only report more stress, emotional and physical traumatic experiences than those with EM, but also show more problems in identifying feelings according to an alexithymia subscale [129]. These findings indicate that CM, rather than EM, is associated with specific personality traits. This does not mean that chronicity triggers personality disorders; however, it is important that clinicians pay attention to personality traits in CM as they may significantly interfere with the treatment.

### Substance use behavior/disorders

Migraine and specific substance use may commonly co-occur. For instance, early epidemiological research found that nicotine dependence and illicit drug use was more frequent among patients with migraine compared to those without; however, a review pointed out, that the association may be more complex [117, 148]. More recent data indicate that the association between migraine and substance use was present only in patients with comorbid depression or PTSD [17, 149]. Thus, substance use may be considered a consequence of other comorbid psychiatric conditions [8].

Higher caffeine consumption could be a risk factor for migraine transformation [150]; indeed, a population-based study found that patients with chronic daily headache were significantly more likely to have been higher caffeine users compared to healthy control subjects [151]. Interestingly, the HEAD-Hunt study found that higher caffeine consumption was positively correlated with infrequent headache and negatively correlated with frequent headache [152], suggesting that either patients with frequent headache avoid caffeine or high-caffeine consumption acts exerting analgesic properties. So far, there is no unequivocal conclusion.

Studies suggest that alcohol consumption is equally or less prevalent in patients with migraine as compared to the general population; possibly, patients self-restrict alcohol consumption because of its actual or assumed action as a trigger of migraine attacks [23, 35, 153–155]. Yet, there is still uncertainty about the mechanisms by which alcohol triggers migraine attacks [156].

### Somatoform disorders/somatic symptoms disorder

Whether somatoform disorders are associated with migraine is generally a matter of debate. According to a literature review, patients with EM without other psychiatric comorbidities display a comparable prevalence of somatoform disorders when compared to non-headache patients [117].

A cross-sectional study performed in a primary headache center found that, among patients with CM, somatic symptoms were significantly more frequent than in patients with EM, while during the follow-up a decrease in somatic symptoms was highly associated with a decrease in headache frequency [157]. In line with these results, the Primary Care Evaluation of Mental Disorders (PRIME-MD) study found that, when compared to patients with episodic headaches, patients with CM had a higher rate of somatic symptoms which increased, together with headache frequency, the likelihood to develop a psychiatric comorbidity [158]. Finally, children with migraine equivalents tend to have more somatic complaints (see above, [145]) and feel more fearful and shy when compared to those without migraine equivalents [159]. However, all those findings are related to somatic symptoms rather than to definite somatoform disorders.

### Eating disorders

Whether eating disorders are linked to migraine is quite controversial. A Finnish study found that, in women with anorexia nervosa or bulimia nervosa, the prevalence of migraine was almost 2-fold higher compared to women without eating disorders (22% vs. 11%); however, a further analysis revealed that the association between eating disorders and migraine was mediated by depression [160].

Thus, eating disorders may enhance the likelihood to develop migraine in specific subgroups of subjects, possibly through the influence of other factors such as anxiety or depression. If migraine patients present themselves with low weight or rapid weight changes, therapists should pay attention to depressive symptoms, given the association between eating disorders and depression. Moreover, specific manifestations of eating disorders, such as dieting, fasting or skipping meals, are often reported as migraine triggers [35, 155].

## Discussion

Overall, our review underlines the consistent association between migraine and psychiatric disorders (e.g., major depression, bipolar disorder, anxiety disorders, PTSD, and other mental disorders including substance-related behavior and personality disorders) that might be attributed to common etiological (both environmental or genetic) factors or to the existence of bidirectional relations between the disorders, as for major depression and panic disorder. Although the actual nature of this complex association between neurological and mental disorders is difficult to determine given the available studies, for therapists it is important to recognize it and to include it into the diagnostic and therapeutic process [161] (see Table 2). This also requires a more intense collaboration between the disciplines of neurology and psychiatry, which are mostly taught separately in medical schools.

**Table 2:** Summary of mechanisms and implications for therapy

Disorder	Possible Mechanisms	Implications for treatment	
		Potential Benefits	Caveats (and potential antidotes)
Depression	- Heritability - Genes (e.g. 5-HT transporter gene, D2 receptor gene) - Neurotransmitter systems (serotonin, dopamine, GABA) - HPA axis - “neuro-limbic” pain network	- Effects of serotonin agonists in both disorders - Specific antidepressants are recommended for migraine and depression (e.g., amitriptyline) - Specific migraine agents can have positive effects for migraine and depression (e.g., onabotulinum toxin A) - Combined pharmacotherapy and psychotherapy can have synergistic effects - Psychotherapy is recommended for migraine and depression (could help to increase adherence to pharmacotherapy or help to use less / no pharmacotherapy)	- Flunarizine and beta-blockers are contraindicated for depression (diagnostic procedures should always include diagnosing for depression) - Patients may not speak about it because of fearing stigma / shame (therapist should try to create an appreciative atmosphere) - Antidepressants recommended for migraine and depression differ in optimal dose for each treatment (weighing of benefits and risks)
Bipolar disorder	- Heritability - Neurotransmitter systems (serotonin, dopamine, glutamate) - Alterations in sodium/calcium channels, pro-inflammatory cytokines	- Effects of antiepileptic drugs in both disorders - Valproate and topiramate (lamotrigine?) can have positive effects for migraine and BD - Psychotherapy is recommended as addition to pharmacotherapy in BD (could help increasing adherence to pharmacotherapy)	- SSRIs and SNRIs have the risk of exacerbating mania or initiating a more rapid cycling course (diagnostic procedures should always include diagnosing for [hypo]manic symptoms, also in family history) - Manic episodes may result in risky behavior (i.e., not taking medication)
Anxiety Disorders	- Heritability - Neurotransmitter systems (serotonin, GABA) - Ovarian hormones	- CBT recommended for migraine and anxiety disorders	- Patients may show avoidant behavior and be skeptical about treatment options - Patients may not speak about anxiety due to several reasons, e.g., subthreshold levels (Therapist should be aware of subthreshold symptoms)
Stress and PTSD	- Central sensitization - Neurotransmitter systems (serotonin)	- CBT (especially stress management) recommended for migraine and stress-related disorders	- Patients may not speak about previous traumatic events
Personality disorders	- ?	- ?	- Personality disorders seem to negatively influence treatment outcome (personality should be considered an influencing factor)
Substance use behavior / disorders	- Depression and other comorbid disorders as associated disorder	- Managing substance use might prevent MOH	- Migraine could be associated with more liberal medication intake (diagnostic procedures should always cover questions on substance use)
Somatoform disorders	- ?	- Reduction in headache may be accompanied by a decrease in somatic symptoms	- Somatic symptoms may complicate treatment (e.g., avoidance behavior)
Eating disorders	- Depression as associated disorder	- For specific subgroups, treating the eating disorder (i.e., avoid fasting, skipping meals, etc.) could reduce headache symptoms	- Eating disorders may be characterized by specific behavior (i.e., avoid fasting, skipping meals, etc.) that may trigger migraine (diagnostic procedures should always cover questions on potential triggers) - Eating disorders are often linked to depression (diagnostic procedures should always include diagnosing for depression) - Patients may not speak about it because of fearing stigma / shame and may hide it with clothes (therapist should be perceptive for eating disorder symptoms)

*5-HT* serotonin, *BD* bipolar disorder, *D2 receptor* dopamine D2 receptor, *GABA* gamma-Aminobutyric acid, *HPA axis* hypothalamic-pituitary adrenal axis, *PTSD* post-traumatic stress disorder, *SNRIs* serotonin–norepinephrine reuptake inhibitors, *SSRIs* selective serotonin reuptake inhibitors

The present systematic review should be considered in the light of the following limitations/shortcomings. For instance, most studies included in the present review used cross-sectional designs, limiting the assessment of causal relations between phenomena (e.g., [161]). Besides, the distribution of sex, age, and migraine subtype, as well as methods to diagnose migraine and psychiatric disorders were largely heterogeneous among the different studies, limiting the possibility of quantifying the real impact of comorbidities in the general population (for more details, see Table 1). Moreover, there are few and mostly indirect data examining the neural mechanisms underlying the comorbidity between migraine and psychiatric comorbidities. Having more than only one psychiatric comorbidity (e.g., concurrent anxiety disorder and major depression) poses another caveat which is often not sufficiently considered in existing studies, but may have further consequences on the course and treatment of migraine [25, 120, 160]. All the mentioned criticisms once again enhance the complexity of migraine and psychiatric comorbidities regarding etiology, pathophysiology or interactions over time. However, the more consistent implications from the reviewed studies have been summarized in Table 2.

Major affective and anxiety disorders have been shown to be the most frequent and disabling psychiatric comorbidities associated with migraine, influencing its clinical course, treatment response, and clinical outcome. Here, the comorbidity has important clinical and therapeutic implications, demanding specific attention by practitioners. For instance, having a comorbid major depression or anxiety disorder can increase the likelihood of suicide attempts in patients with migraine [20]. The comorbidity with psychiatric disorders is not uniformly increased in the different migraine subtypes, but generally more elevated in patients with CM or migraine with aura, suggesting the need for more specific care in those patients.

Genetic variants, dysfunction in neurotransmitters (especially 5-HT), and HPA axis dysregulation are among the most supported pathophysiological mechanisms underlying the comorbidity between migraine and depression. Specific neural network patterns overlap between both entities, which may be the result of the above hypothesized mechanisms. Unfortunately, no specific and valid biomarkers have been documented for the risk of comorbid migraine and depression. Still, it is not sufficiently clear, if the observed biological or chemical parameters are indicators of specific underlying etiological and pathogenic pathways or if they represent epiphenomena.

The relation between migraine and major depression is likely to be bidirectional. Regarding the bipolar spectrum, clinicians need to suspect and identify the possible comorbidity between migraine and BD, especially among female patients and subjects with BD II. Here, the possible misidentification of unipolar depressive subtypes should be avoided, as pharmacological indications for the two conditions widely differ or may be counterproductive.

Whether the comorbidity between migraine and anxiety is uni- or bidirectional is a matter of debate. Excess worry, fear, and other anxiety symptoms such as avoidance behavior are usually part of the clinical migraine presentation, while, conversely, headache symptoms may also be part of a clinical anxiety disorder. This implies the need to correctly identify the clinical characteristics of both these conditions, to derive a working hypothesis regarding which of the disorders may be primary or secondary, as the adequate treatment of the primary condition may be beneficial for the outcome of the secondary condition. PD appears to be more consistently associated with migraine than most other anxiety disorders. Comorbid PD is associated with greater health care costs, higher disability, and functional impairment as well as risk for chronification, medication overuse, and henceforth MOH. As with migraine and depression, the relation between PD and migraine appears to be bidirectional. The link between PTSD and migraine seems to be more evident in chronic forms, as the repeated exposure to stressful situations/factors causes cortical response modification with modulation of the vascular trigeminal system leading to a lower pain threshold.

There are few and sometimes conflicting studies in the current literature concerning the comorbidity between migraine and psychiatric disorders associated with minor incidence in the general population, including personality disorders, substance use disorders, somatoform disorders, and eating disorders. Here, the association may be also due to or mediated by concurrent major depression. Specific heed should be paid to MOH, which is more common in patients with comorbid psychiatric disorders [162]. In this condition, pain-relieving drugs for the acute treatment of headache symptoms may themselves lead to headache when taken frequently for long periods. Here, therapists should explain this phenomenon in detail to the patients in order to assure a proper use of prescribed or over-the-counter medication. In some cases of MOH, comorbid psychiatric symptoms might be considered an epiphenomenon of medication overuse – at least animal data imply such a mechanism [163].

**Fig. 2:**
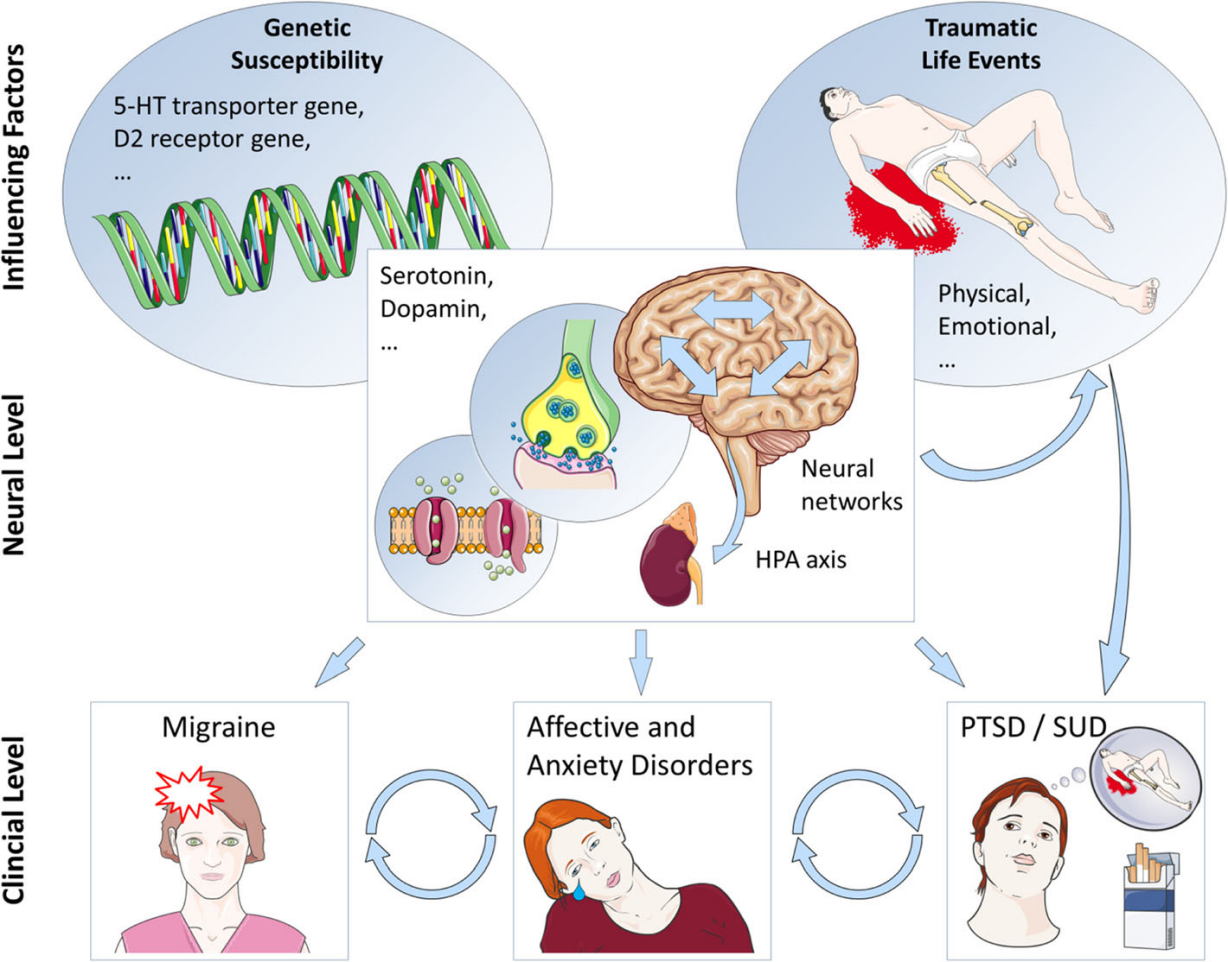
Scheme of the association mechanisms between migraine and psychiatric disorders. The Figure summarizes the mechanisms potentially involved in the comorbidity of migraine and psychiatric disorders on different levels. Shared genetic susceptibility and traumatic life events can be considered important influencing factors. On the neural level, cellular changes (channels), neurohormonal changes (HPA axis), neurotransmitter changes (serotonergic, dopaminergic, and glutamatergic neural pathways) and neural network changes are discussed. On the clinical level, migraine co-occurs with different manifestations of psychiatric disorders (for abbreviations see below)

Reviewing the current literature, we are still far from comprehensively understanding the biological origin/axis underlying the migraine and psychiatric disorder comorbidity. For most of the comorbid disorders, the commonly mentioned pathogenic players such as heritability, specific genes or neurotransmitter systems may play a significant role (Fig. 2). As influences seem to be generally complex and dynamic in their nature over time, simple therapeutic solutions are not available and combined approaches are required. Instead, the involvement of different disciplines is needed in order to carefully account for each patient’s disorder and treatment history in a multimodal treatment approach perspective.

Clarifying the comorbidity between psychiatric disorders and migraine is essential not only at the clinical diagnostic level but above all for the complex therapeutic implications of such comorbidity [164]. CBT has been shown in several population studies to be a valid alternative in addition to pharmacological treatments in patients with migraine and psychiatric comorbidity. Importantly, the pharmacological prophylaxis of migraine might be influenced by psychiatric comorbidities. Many biological and neural aspects related to the comorbidity still need to be clearly elucidated to better approach the real complexity of this issue.

## Conclusion

Our intention is to conclude this review emphasizing three most relevant key points for clinicians:

1. Psychiatric comorbidity in migraine is common and invalidating.
2. The careful history taking and diagnostic procedures related to migraine should carefully take into account the existence of comorbidities.
3. Migraine management and treatment should be tailored to consider the presence of psychiatric comorbidities, taking into account the potential beneficial or synergistic effects as well as treatment complications.

## Abbreviations

5-HT: Serotonin
5-HTTLPR: Serotonin-transporter-linked polymorphic region
BD: Bipolar disorder
CBCL: Child Behavior Checklist
CBT: Cognitive-behavioral therapy
CGRP: Calcitonin gene-related peptide
CM: Chronic migraine
D2 receptor: Dopamine D2 receptor
EM: Episodic migraine
GABA: Gamma-Aminobutyric acid
GAD: Generalized anxiety disorder
HPA axis: Hypothalamic-pituitary adrenal axis
HR: Hazard ratio
MMPI-2: Minnesota Multiphasic Personality Inventory
MOH: Medication-overuse headache
OCD: Obsessive-compulsive disorder
OR: Odds ratio
PD: Panic disorder
PR: Prevalence ratio
PRIME-MD: Primary Care Evaluation of Mental Disorders
PRISMA: Preferred Reporting Items for Systematic Reviews and Meta-Analyses
PTSD: Post-traumatic stress disorder
RR: Relative risk
SNRIs: Serotonin–norepinephrine reuptake inhibitors
SSRIs: Selective serotonin reuptake inhibitors
SUD: Substance use disorder
